# Multipartite Entanglement Generation in a Structured Environment

**DOI:** 10.3390/e22020191

**Published:** 2020-02-07

**Authors:** Shijiao Wang, Xiao San Ma, Mu-Tian Cheng

**Affiliations:** School of Electric Engineering and Information, Anhui University of Technology, Ma’anshan 243002, China; wangshijiao328@163.com

**Keywords:** multipartite entanglement, entanglement generation, a structured environment, 03.65.Ud, 03.67.Mn

## Abstract

In this paper, we investigate the entanglement generation of *n*-qubit states in a model consisting of *n* independent qubits, each coupled to a harmonic oscillator which is in turn coupled to a bath of *N* additional harmonic oscillators with nearest-neighbor coupling. With analysis, we can find that the steady multipartite entanglement with different values can be generated after a long-time evolution for different sizes of the quantum system. Under weak coupling between the system and the harmonic oscillator, multipartite entanglement can monotonically increase from zero to a stable value. Under strong coupling, multipartite entanglement generation shows a speed-up increase accompanied by some oscillations as non-Markovian behavior. Our results imply that the strong coupling between the harmonic oscillator and the *N* additional harmonic oscillators, and the large size of the additional oscillators will enhance non-Markovian dynamics and make it take a very long time for the entanglement to reach a stable value. Meanwhile, the couplings between the additional harmonic oscillators and the decay rate of additional harmonic oscillators have almost no effect on the multipartite entanglement generation. Finally, the entanglement generation of the additional harmonic oscillators is also discussed.

## 1. Introduction

Multipartite entanglement is of significance for quantum information processing such as quantum communication, quantum teleportation, quantum cryptography, and so on. Much work concerning multipartite entanglement has been done recently, on many aspects [1,2,3,4,5,6,7,8,9,10,11,12,13,14,15,16,17,18,19,20,21,22,23,24,25,26,27,28,29,30,31,32,33,34,35,36]. One important problem concerning multipartite entanglement is the entanglement generation. Researchers have paid a great deal of attention, both experimentally and theoretically, to multipartite entanglement generation, and many results have been obtained [37,38,39,40,41,42,43,44,45,46,47,48]. In these works, the effect induced by the environment on the multipartite entanglement is an interesting problem deserving study. Usually, the decoherence [49] will destroy the entanglement due to the system–environment interaction and the preservation of entanglement significant for quantum information processing. Therefore, avoiding the interaction with the environment is necessary to preserve the quantum entanglement as much as possible. Recently, some works have proposed achieving long-term entangled states via coupling qubits to a common and dissipative environment [50,51,52,53,54,55,56,57,58]. In these works, the environment plays a constructive role in generating entangled states of the system, even though the parties of the system have no interaction.

In fact, the role of the environment is divided into memory effects and memoryless effects. The memoryless environment allows the information to flow only in a direction from the system to the environment. The Markovian dynamics appears here. In contrast, a memory environment will make the information flow back from the environment to the system, and this leads to non-Markovian dynamics. Non-Markovian dynamics has attracted a great deal of attention from researchers in both theoretical and experimental studies because of its important role in quantum state engineering, quantum control, and quantum information processing [59,60,61,62,63,64,65,66,67,68,69,70,71,72,73,74,75,76,77,78,79,80,81,82]. One important aspect of non-Markovian dynamics is its effect on the entanglement generation of the system’s quantum states. In this paper, we consider a model consisting of an *n*-qubit quantum system whose qubits are independent and couple to a harmonic oscillator, which is in turn coupled to a bath of *N* additional harmonic oscillators interacting themselves with nearest-neighbor coupling. Similar realistic modeling of such a system has been studied for the temperature crossover of decoherence rates of a quantum system interacting with many harmonic oscillators [67]. In this paper, the harmonic oscillator interacting with the *n* independent qubits directly channels the flux of coherence between the system of interest and a complex bath. The additional harmonic oscillators have no direct interaction with the qubits of interest but couple to the harmonic oscillator and contain nearest-neighbor coupling between themselves. This model is employed by Zhang’s group [72] to investigate speed-up dynamics control of a quantum system in a hierarchical environment. This model is similar to the model proposed by Ting Yu’s group [66], where the authors analyzed the crossover from the Markovian dynamics to non-Markovian dynamics. Note that an experimental study concerning non-Markovian dynamics for the same qubit-cavity model was made carefully in [83]. In this paper, we want to study the entanglement evolution of the *n*-qubit states and consider the entanglement generation between the qubits of the system. Specifically, the effect of model parameters on the time evolution of the quantum entanglement is considered in order to obtain an understanding of the interesting features.

The rest of this paper is organized as follows: In Section 1, we introduce the model describing our system. In Section 2, we investigate the entanglement dynamics of quantum states initially taking a separable state to analyze the multipartite entanglement generation. Finally, conclusions of our results are given.

## 2. Hamiltonian of Our Model

**Figure 1:**
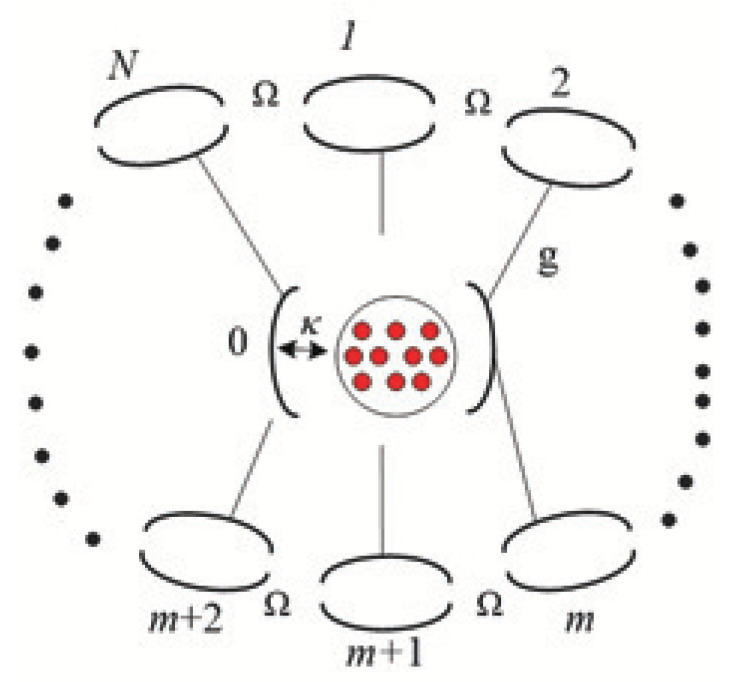
*n* independent qubits (red) interact with the 0-th harmonic oscillator, which couples to the *N* additional harmonic oscillators that interact between themselves via nearest-neighbor coupling.

We consider the following model which consists of an *n*-qubit quantum system with a structured environment consisting of one harmonic oscillator labeled by 0 and *N* additional harmonic oscillators labeled by *m*(m=1,2,⋯,N), as depicted in Figure 1.

This model is an extension of a system with one qubit [72] to a system with *n* qubits to consider the multipartite entanglement generation. The total Hamiltonian is given as $H=H_{0}+H_{I}$, and $H_{0},H_{I}$ read

(1) $$H_{0}=\sum_{i=1}^{n}\frac{\omega_{s}}{2}\sigma_{i}^{z}+\omega_{0}a^{†}a+\sum_{m=1}^{N}\omega_{m}b_{m}^{†}b_{m}$$

(2) $$H_{I}=\sum_{i=1}^{n}\kappa \left(\sigma_{i}^{+}a+\sigma_{i}^{-}a^{†}\right)+\sum_{m=1}^{N}g\left(ab_{m}^{†}+a^{†}b_{m}\right)+\sum_{\langle jk\rangle }\Omega \left(b_{j}^{†}b_{k}+b_{k}^{†}b_{j}\right)$$

where $\omega_{s}$ are the frequencies of the qubits of the quantum system. $\omega_{0}\;\mathrm{and}\;\omega_{m}$ are the frequencies of the 0th harmonic oscillator and *N* additional harmonic oscillators, respectively. For convenience, we let $\omega_{s}=\omega_{0}=\omega_{m}$ as they bring about free evolution. $\sigma_{i}^{z}$ is the familiar Pauli matrix of the *i*th qubit. $\sigma_{i}^{\pm }$ are the raising and lowering operators of the *i*th qubit of the quantum system. $a^{†}\left(a\right)$ and $b_{m}^{†}\left(b_{m}\right)$ are the corresponding creation (annihilation) operators for the 0th harmonic oscillator and the *N* additional harmonic oscillators, respectively. κ is the coupling between the quantum system and the 0th harmonic oscillator, *g* is the coupling between the 0th harmonic oscillator and the *N* additional harmonic oscillators, and Ω is the coupling between the nearest harmonic oscillators of the complex bath. 〈jk〉 means the nearest-neighbor harmonic oscillators of the complex bath. If the decay of all the harmonic oscillators is taken into account, we get the following master equation for the density matrix ρ(t) [72].

(3) $$\frac{d\rho }{dt}=-i\left[H,\rho \right]-\frac{\Gamma_{0}}{2}\left(a^{†}a\rho -2a\rho a^{†}-\rho a^{†}a\right)-\sum_{m=1}^{N}\frac{\Gamma }{2}\left(b_{m}^{†}b_{m}\rho -2b_{m}\rho b_{m}^{†}-\rho b_{m}^{†}b_{m}\right)$$

where $\Gamma_{0}$ and Γ are the decay rates of the 0th harmonic oscillator and the *N* additional harmonic oscillators, respectively. Our focus is on the effects of parameters g,Ω of the coupling strengths, the size *n* of the quantum system, and the number *N* of the additional oscillators on the entanglement generation of the quantum system. The analytical solution of the above master equation is difficult to obtain. Fortunately, we can use the Schrödinger equation to get the approximate solution to the above master equation. In order to take the dissipative effect of all the oscillators into account, we add two terms of non-Hermitian Hamiltonian of $-i\frac{\Gamma_{0}}{2}a^{†}a-\sum_{m=1}^{N}i\frac{\Gamma }{2}b_{m}^{†}b_{m}$ as the approximate dissipative effect of all the oscillators to the effective Hamiltonian $H_{eff}$ which ignore the free evolution of Hamiltonian $H_{0}$. We can use the Schrödinger equation to study the time evolution of quantum states of concern,

(4) $$|\dot{\psi }\left(t\right)\rangle =-iH_{eff}|\psi \left(t\right)\rangle$$

Here, we assume the initial state of all the oscillators is the vacuum state and the initial state of the quantum system is in an arbitrary superposition of single excitation of qubits. Note that the initial state of the quantum system can be a vacuum state and the initial state of all the oscillators is in a single-photon state. This assumption can also be analyzed in detail, and will be discussed in the following content. It should be noted that the analytical solutions to the time-dependent Schrödinger equation are hard to find with arbitrary initial state. If higher excitation is taken into account, it is difficult to get the determined resulted state due to the complicated calculation.

## 3. Time Evolution of Quantum States

Usually, the time evolution of quantum states is hard to find for the arbitrary initial state. In this paper, we consider that the initial state of the quantum system is a state with only one qubit in the excited level, and all the other qubits are in the ground state. The initial state of all the oscillators is in a vacuum state without any photon. Because the Hamiltonian makes the particle number conserved, the resulted state will contain one excitation at any time during the evolution. The initial state of the composite system consisting of an *n*-qubit system and the structured environment reads $\left|\psi \left(0\right)\rangle =\right|1_{x},0_{0},0_{m}\rangle$, where $|1_{x}\rangle$ denotes the initial state of the *n*-qubit system with the *x*th qubit being in an excited level and all the other qubits being in ground state, and $|0_{0}\rangle ,|0_{m}\rangle$ are the initial states as vacuum states of all the oscillators of the structured environment, respectively. Obviously, the initial state of the composite state is a separable state. According to the Schrödinger equation with the expression of Equation (4), we can get the total resulted state in the following form as in [54,72]:

(5) $$|\psi \left(t\right)\rangle =C_{0}\left(t\right)|1_{x}\rangle |0_{0},0_{m}\rangle +C_{1}\left(t\right)|W_{n-1},0_{0},0_{m}\rangle +C_{2}\left(t\right)|0,1_{0},0_{m}\rangle +\sum_{m}C_{m}\left(t\right)|0,0,1_{m}\rangle$$

where $|1_{0}\rangle$ is the excited state of the 0th harmonic oscillator, $|1_{m}\rangle$ denotes that the state of the *N* additional harmonic oscillators with the *m*th harmonic oscillator being in an excited state and all the other oscillators being in the ground state. The state of $|W_{n-1}\rangle$ reads $|W_{n-1}\rangle =\frac{1}{\sqrt{n-1}}\sum_{i\neq x}|1_{i}\rangle$. $C_{q}\left(t\right)\left(q=0,1,2,m\right)$ are the coefficients satisfying the following equations. Note that the coefficients of $C_{m}\left(t\right)\left(m=1,\cdots ,N\right)$ take the same expression due to the symmetry and we denote them as C(t). From Equation (4) and the form of the resulted state, we get the following differential equations:

(6) $$\begin{array}{ccc} {\dot{C}}_{0}\left(t\right) & = & -i\kappa C_{2}\left(t\right) \end{array}$$

(7) $$\begin{array}{ccc} {\dot{C}}_{1}\left(t\right) & = & -i\kappa \sqrt{n-1}C_{2}\left(t\right) \end{array}$$

(8) $$\begin{array}{ccc} {\dot{C}}_{2}\left(t\right) & = & -i\kappa C_{0}\left(t\right)-i\kappa \sqrt{n-1}C_{1}\left(t\right)-\frac{\Gamma_{0}}{2}C_{2}\left(t\right)-igNC\left(t\right) \end{array}$$

(9) $$\begin{array}{ccc} \dot{C}\left(t\right) & = & -igC_{2}\left(t\right)-i2\Omega C\left(t\right)-\frac{\Gamma }{2}C\left(t\right) \end{array}$$

It is difficult to get the analytical expression of the time evolution of the quantum state. However, we can use the Laplace transform to get the resulting time evolution by numerical simulation. The initial conditions are listed as $C_{0}\left(0\right)=1,C_{1}\left(0\right)=C_{2}\left(0\right)=C\left(0\right)=0$. The expressions of the Laplace transforms of $C_{0}\left(t\right),C_{1}\left(t\right),C_{2}\left(t\right)$, C(t) are given by

(10) $$C_{0}\left(p\right)=\frac{1}{p}-\frac{\kappa^{2}}{p\left(p^{2}+\frac{\Gamma_{0}p}{2}+n\kappa^{2}+\frac{pg^{2}N}{p+i2\Omega +\frac{\Gamma }{2}}\right)}$$

(11) $$C_{1}\left(p\right)=\frac{-\kappa^{2}\sqrt{n-1}}{p\left(p^{2}+\frac{\Gamma_{0}p}{2}+n\kappa^{2}+\frac{pg^{2}N}{p+i2\Omega +\frac{\Gamma }{2}}\right)}$$

(12) $$C_{2}\left(p\right)=\frac{-i\kappa }{p^{2}+\frac{\Gamma_{0}p}{2}+n\kappa^{2}+\frac{pg^{2}N}{p+i2\Omega +\frac{\Gamma }{2}}}$$

(13) $$C\left(p\right)=\frac{-g\kappa }{\left(p+i2\Omega +\Gamma /2\right)\left(p^{2}+\frac{\Gamma_{0}p}{2}+n\kappa^{2}+\frac{pg^{2}N}{p+i2\Omega +\frac{\Gamma }{2}}\right)}$$

For convenience, we choose $C_{2}\left(p\right)$ to analyze the dynamics of the parameters. Then, $C_{2}\left(t\right)=L^{-1}\left[C_{2}\left(p\right)\right]$, and the other parameters $C_{0}\left(t\right),C_{1}\left(t\right),\;\mathrm{and}\;C\left(t\right)$ can be obtained similarly.

In order to get some understanding of the time evolution of $C_{2}\left(t\right)$, we will make some discussion of the expression of the $C_{2}\left(p\right)$. At first, we assume that the coupling strength between the 0th harmonic oscillator and the *N* additional harmonic oscillators is weak enough. Under very weak coupling *g* of the harmonic oscillators, the term of $\frac{pg^{2}N}{p+i2\Omega +\frac{\Gamma }{2}}$ can be ignored for the number of *N* taking a small value. Then, we can get the analytical expression of the time evolution of $C_{2}\left(t\right)$ in the following expression.

(14) $$C_{2}\left(t\right)=\frac{-4i\kappa }{\Lambda }\exp\left(\frac{-\Gamma_{0}t}{4}\right)\sinh\left(\frac{\Lambda t}{4}\right)$$

where $\Lambda =\sqrt{\Gamma_{0}^{2}-16n\kappa^{2}}$ when κ satisfies the relation $\kappa \leq \frac{\Gamma_{0}}{4\sqrt{n}}$. For this condition of weak coupling between the *n*-qubit system and the 0th harmonic oscillator, we can find that the time evolution of $C_{2}\left(t\right)$ takes a monotonic evolution and the dynamics corresponds to Markovian dynamics. For the strong coupling of the relation $\kappa \geq \frac{\Gamma_{0}}{4\sqrt{n}}$, we get the time evolution of $C_{2}\left(t\right)$ in the following expression:

(15) $$C_{2}\left(t\right)=\frac{-4i\kappa }{\Lambda_{0}}\exp\left(\frac{-\Gamma_{0}t}{4}\right)\sin\left(\frac{\Lambda_{0}t}{4}\right)$$

where $\Lambda_{0}=\sqrt{16n\kappa^{2}-\Gamma_{0}^{2}}$. The expression of $C_{2}\left(t\right)$ in Equation (15) implies that the time evolution corresponds to the non-Markovian dynamics for strong coupling between the *n*-qubit system and the 0th harmonic oscillator. This formally reproduces the results in [68,84] with little difference in the size of the system.

When the parameters g,N satisfy the relation that $g^{2}N$ takes a sufficiently large value, the expression of $C_{2}\left(p\right)$ will become the following expression:

(16) $$C_{2}\left(p\right)=\frac{-i\kappa }{p^{2}+\frac{\Gamma_{0}p}{2}+n\kappa^{2}+g^{2}N}$$

Comparing Equation (16) with Equation (12), we can find that the constant term changes from $n\kappa^{2}$ to $n\kappa^{2}+g^{2}N$. The large value of coupling strength *g* between the 0th harmonic oscillator and the *N* additional harmonic oscillators, as well as the size of additional harmonic oscillators, will contribute to the coupling strength between the system and the 0th harmonic oscillator to some extent. Therefore, the time evolution of $C_{2}\left(t\right)$ is affected by the parameters κ,n,g,N,Γ commonly. The roles played by the parameters of the model should be examined in detail to analyze the multipartite entanglement generation. We especially want to understand the effect of the *N* additional harmonic oscillators on the multipartite entanglement to find appropriate ways to control the entanglement dynamics of the quantum system indirectly.

The above analysis can also be applied to the parameters $C_{0}\left(t\right),C_{1}\left(t\right)$, and we can find that the coupling strengths κ,g,Ω and the sizes n,N are responsible for the Markovian dynamics and non-Markovian dynamics of the quantum system. Taking a further look into Equation (12), one can find that the *N* additional harmonic oscillators can affect the time evolution of quantum states of the *n*-qubit quantum system without any approximation, even though the *N* additional harmonic oscillators have no direct interaction with the *n*-qubit quantum system. The *N* additional harmonic oscillators can induce the Markovian and non-Markovian dynamics of the *n*-qubit quantum system. The analytical expression of $C_{2}\left(t\right)$ as the inverse of $C_{2}\left(p\right)$ in Equation (12) could not be obtained, but we can use the numerical simulation instead to investigate the entanglement generation of the *n*-qubit system in the following content.

## 4. Multipartite Entanglement Generation

In order to analyze the multipartite entanglement dynamics of the quantum state of the *n*-qubit system, we should introduce the multipartite entanglement measure and we choose the linear entropy proposed by the authors in [85] as the multipartite entanglement measure. As the linearized version of the von Neumann entropy, the linear entropy is used to ease theoretical calculations and it works very well for such a W-type state in Equation (5). The linear entropy is defined as

(17) $$Q\equiv \left[\frac{1}{n}\left(\sum_{i=1}^{n}L_{i}\right)\right]$$

where *n* is the size of the system. $L_{i}$ is the linear entropy of the *i*th qubit and the linear entropy is expressed as $L_{i}=2\left[1-\mathrm{Tr}\left(\rho_{i}^{2}\right)\right]$. Since the linear entropy of one subsystem of a pure state is an entanglement monotone [86] (it does not increase under local operations and classical communication), and is a valid entanglement measure. A concave function of the linear entropy is also an entanglement monotone. Therefore, the average linear entropy *Q* is an entanglement monotone and can be employed to study the multipartite entanglement generation. Here, in slight contrast to the definition in [85], we consider only the multipartite entanglement of the *n*-qubit system and do not take the linear entropy of the environment into account because the multipartite entanglement of the *n*-qubit system should be examined independently. For instance, given a W-type state with a size of 5, the linear entropy takes a value of 0.4. Through calculation, we get the linear entropy of the *n*-qubit quantum states by tracing the degrees of freedom of the structured environment off the composite state in Equation (5) in the following expression:

(18) $$Q=\frac{4}{n}|C_{1}{\left(t\right)|}^{2}-\frac{4}{n(n-1)}{\left|C_{1}\left(t\right)\right|}^{4}+\frac{4}{n}\left(|C_{0}{\left(t\right)|}^{2}-{\left|C_{0}\left(t\right)\right|}^{4}\right)$$

Obviously, the linear entropy depends on the parameters of the system size *n*, $C_{1}\left(t\right)$, and $C_{0}\left(t\right)$. $C_{1}\left(t\right)$ and $C_{0}\left(t\right)$ can be derived from $C_{2}\left(p\right)$ in Equations (10)–(13) with Laplace transform. The linear entropy depends on the system size *n*, the coupling strengths κ,g,Ω, and the dissipative effect of Γ. Next, we will examine the effect of the coupling strengths including κ, *g*, Ω, and the sizes of the system and the environment n,N on the linear entropy.

**Figure 2:**
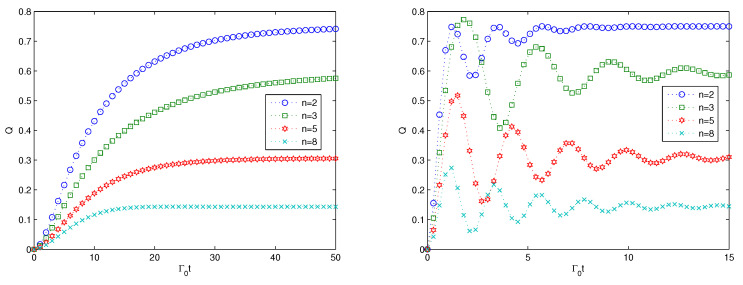
Multipartite entanglement evolution versus the scaled time $\Gamma_{0}t$ is plotted for the weak coupling $\kappa =0.1\Gamma_{0}$ on the (**left**) and for the strong coupling $\kappa =\Gamma_{0}$ on the (**right**), respectively. Here, N=3,g=0.1κ,Γ=0.1κ,Ω=0.1κ.

First, we consider the effect of the size of the system *n* on the linear entropy for the cases of weak coupling and strong coupling in the two subfigures of Figure 1, respectively. From the left subfigure in Figure 2 for the weak coupling, we find that the multipartite entanglement can be generated as the linear entropy increases from zero to stable values for different sizes of the quantum system. The larger the size of the quantum system is, the smaller the stable value is. Due to the definition of the linear entropy as the average linear entropy, the stable value for the system with a large size is a small value. Such a small value does not mean that the multipartite entanglement is very small, as the average entropy will lead to the value of multipartite entanglement taking a small value for the quantum states with a large size. For the strong coupling in Figure 2, we can find that the multipartite entanglement will increase very rapidly to the maximum value, then demonstrate some oscillations and reach stable values for different sizes of the quantum system. The stable values for the strong coupling are the same as with the case of the weak coupling. Compared with the weak coupling, the multipartite entanglement for the strong coupling perceives some oscillations. Such oscillations indicate non-Markovian dynamics. While for the weak coupling, the entanglement increases monotonically from zero to stable values. From the analysis, we can find that either under weak coupling or under strong coupling, the multipartite entanglement can be generated for the *n* qubits and the decay rate of the 0th oscillator does not affect the stable values for the *n*-qubit states but affects the time for the entanglement to reach a stable value. The monotonic increase behavior represents Markovian dynamics. By comparing the two subfigures, we can find that the non-Markovian dynamics can speed up the multipartite evolution of the quantum states, as the linear entropy increases faster initially for the strong coupling than for the weak coupling. This conclusion is consistent with the results in [72], where the author analyzed the time evolution of non-Markovianity.

**Figure 3:**
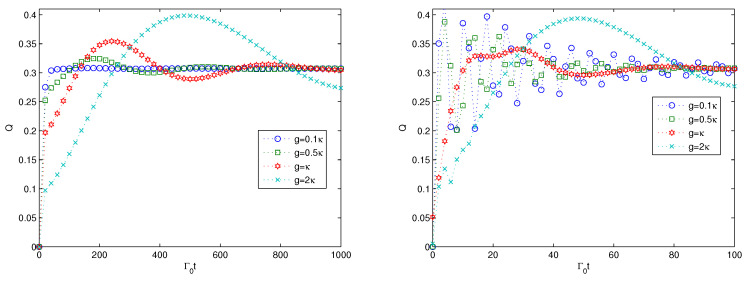
Time evolution of the linear entropy versus the scaled time of $\Gamma_{0}t$ plotted for the weak coupling $\kappa =0.1\Gamma_{0}$ on the (**left**) and for strong coupling $\kappa =\Gamma_{0}$ on the (**right**), respectively. Here, n=5,N=3,Γ=0.1κ,Ω=0.1κ.

Next, we consider the effect of the coupling strength *g* between the 0th harmonic oscillator and the *N* additional harmonic oscillators on the multipartite entanglement generation, and the results are presented in Figure 3. From Figure 3, we can find that the entanglement generation shows interesting behaviors. In the left subfigure, the multipartite entanglement increases monotonically from zero to a stable value for the case of g=0.1κ. With the increase of the value of *g* as the coupling between the 0th harmonic oscillator and the *N* additional harmonic oscillators, the multipartite entanglement increases to stable values with some oscillations even for the weak coupling of $\kappa =0.1\Gamma_{0}$. The larger the coupling strength *g* is, the larger the oscillation is. The oscillating behavior characterizes the non-Markovian dynamics. In this sense, the strong coupling between the 0th harmonic oscillator and the *N* additional oscillators contributes to the non-Markovian dynamics of the multipartite entanglement generation of the quantum system. Comparing the two left subfigures of Figure 2 and Figure 3, we can find that the multipartite entanglement needs a longer time to reach a stable value with larger oscillations for the stronger coupling parameter of *g*. For the strong coupling $\Gamma_{0}=\kappa$ in the right sub-figure, we can find that the multipartite entanglement increases much faster to the maximum value, then oscillates to reach the stable value. The larger the parameter of *g* is, the larger the oscillation is. Comparing the two subfigures in Figure 3, we can find that the quasi-periods are far smaller for the strong coupling in the right subfigure than for the weak coupling in the left subfigure. Additionally, the multipartite entanglement evolution demonstrates a speed-up behavior for the strong coupling in the right subfigure, as the scaled time for the entanglement to reach a large value is far shorter than that of the case in the left subfigure.

**Figure 4:**
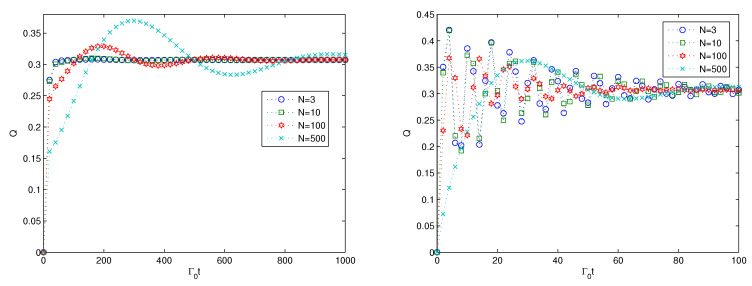
Time evolution of the linear entropy versus the scaled time of $\Gamma_{0}t$ plotted for the weak coupling $\kappa =0.1\Gamma_{0}$ on the (**left**) and for the strong coupling $\kappa =\Gamma_{0}$ on the (**right**), respectively. Here, n=5,g=0.1κ,Ω=0.1κ,Γ=0.1κ.

Next, we investigate the effect of the size *N* of the additional harmonic oscillators on the multipartite entanglement generation, and the results are presented in Figure 4. From the left subfigure in Figure 4, the multipartite entanglement evolution increases to stable a stable value, accompanied by some oscillations. The larger the size of *N* is, the larger the oscillation is, and the larger the quasi-period. The oscillating behavior characterizes the non-Markovian dynamics of the quantum states. For the strong coupling in the left subfigure, the quasi-period is far shorter than that for the weak coupling, and the multipartite entanglement reaches the stable value faster than that for the weak coupling. The results in Figure 4 imply that the large size of the additional harmonic oscillators contributes to the non-Markovian dynamics of the quantum entanglement. The effect of the size of the additional harmonic oscillators is similar to that of the coupling strength between the 0th harmonic oscillator and the *N* additional harmonic oscillators to a large extent due to the term of multiplication $g^{2}N$ in Equations (10)–(13). Therefore, the similar effect of *g* and *N* is not surprising.

**Figure 5:**
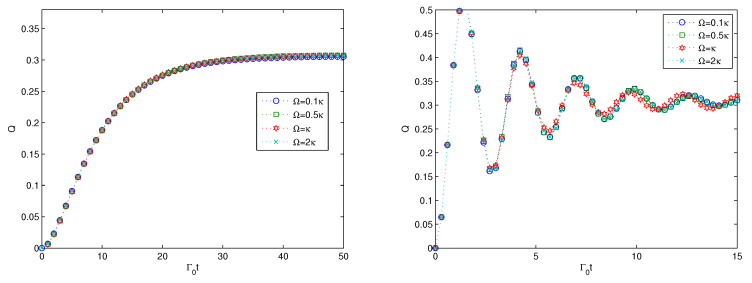
Time evolution of the linear entropy versus the scaled time of $\Gamma_{0}t$ plotted for the weak coupling $\kappa =0.1\Gamma_{0}$ on the (**left**) and for the strong coupling $\kappa =\Gamma_{0}$ on the (**right**), respectively. Here, n=5,N=3,g=0.1κ,Γ=0.1κ.

Finally, we consider the effect of the parameter Ω on the multipartite entanglement generation, and the results are presented in Figure 5. From Figure 5, we can find that the entanglement shows almost the same behavior for various values of Ω. For the weak coupling in the left subfigure in Figure 5, the multipartite entanglement increases monotonically from zero to the stable value of 0.2. The variation of the the parameter Ω imposes no effect on the multipartite entanglement evolution. For the strong coupling case, the cases of the different values of Ω coincide with each other and have no obvious difference. The results of Figure 5 imply that the couplings between the harmonic oscillators via nearest-neighbor coupling has no effect on the multipartite entanglement of the quantum states. Except for the parameter Ω, the parameter Γ to characterize the dissipative effect of the *N* additional harmonic oscillators is also considered, and the results show that it also has no effect on the multipartite entanglement generation. Therefore, we omit the numerical simulation of the effect of the parameter Γ on the multipartite entanglement generation.

**Figure 6:**
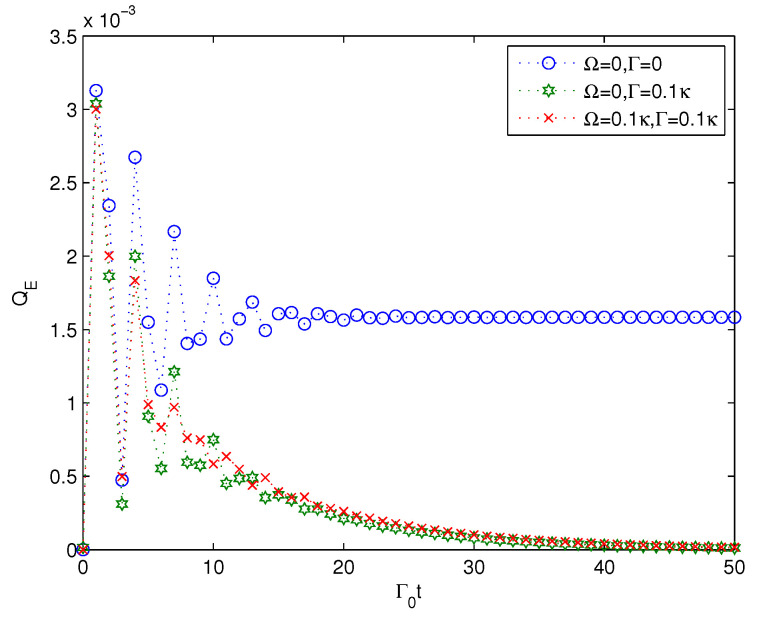
Time evolution of the linear entropy of the cavities of the environment $Q_{E}$ versus the scaled time of $\Gamma_{0}t$ plotted for the strong coupling $\Gamma_{0}=\kappa$. Here, n=5,N=3,g=0.1κ.

In the above content, we analyzed the multipartite entanglement generation of a quantum system interacting with a harmonic oscillator, which in turn couples to *N* additional harmonic oscillators. The analysis is based on the assumption that the initial state of the quantum system is a one-excitation state and the initial state of the structured environment is in a vacuum state. In fact, we only need to preserve the number of excitations of the whole system-environment initial state as one to get the corresponding analysis. Therefore, the case that the initial state of the environment is a single-photon state and that the quantum system is in ground state can also be analyzed similarly. However, our focus is on the multipartite entanglement generation of the quantum system. Therefore, we do not consider the case where the initial state of the environment is in a single-photon state, but consider the entanglement generation of the *N* additional harmonic oscillators under the assumption that the initial state of the quantum system is a one-excitation state instead. This consideration can be a complementary study of the entanglement generation under the case that the initial state of the environment is a single-photon state, and the results of the multipartite entanglement generation of cavities $Q_{E}$ are presented in Figure 6.

From Figure 6, we can find that only when both Ω and Γ take a zero value can the entanglement of $Q_{E}$ reach a stable value. When either Ω or Γ takes a non-zero value, the multipartite entanglement of cavities of the *N* additional harmonic oscillators will increase from zero to a value initially, then decrease to zero after some time. This implies that the multipartite entanglement of the *N* additional harmonic oscillators exists temporarily for the cases of the parameters of Ω and Γ with nonzero values. It should be pointed out that the case of Ω=0.1κ,Γ=0 is considered in the numerical simulation. However, our results show that the corresponding coefficient of $C_{m}\left(t\right)$ in Equation (5) for the case Ω=0.1κ,Γ=0 takes some nonphysical results as the value of $C_{m}\left(t\right)$ is larger than 1 for some time points. Therefore, the case of Ω=0.1κ,Γ=0 is omitted here to maintain consistency with our forward analysis. Even though the multipartite entanglement of the *N* additional harmonic oscillators is generated, its value is so small that it can be ignored to some extent. Therefore, when the initial state of the environment is a single-photon state, the multipartite entanglement generation of the quantum system will similarly be very minor.

Here, it should be noted that our analysis is based on the assumption that there is initially only one excitation in the qubit state. If the initial state of the system is in ground state and the initial state of the environment is in vacuum state, there is no entanglement generation with our model. Therefore, if all the initial states including the qubits and the cavities are in ground state, we should employ the nonlinear interaction [87] to generate multipartite entanglement.

## 5. Conclusions

We investigated the multipartite entanglement generation of a quantum system interacting with a harmonic oscillator which is in turn coupled to *N* additional harmonic oscillators that interact with themselves via nearest-neighbor coupling. With our analysis, we find that the multipartite entanglement can be generated by the interaction between the system and the structured environment. For the weak coupling between the system and the 0th harmonic oscillator, the multipartite entanglement takes a monotonic increasing behavior with the scaled time. For the strong coupling between the quantum system and the harmonic oscillator, the multipartite entanglement shows a speed-up evolution with oscillations characterizing non-Markovian dynamics. Taking the interaction between the 0th harmonic oscillator and the *N* additional harmonic oscillators into account, we find that the strong coupling strength between the 0th harmonic oscillator and the *N* additional harmonic oscillators, and a large number *N* of the additional harmonic oscillators can enhance the non-Markovian dynamics of the multipartite entanglement generation. At the same time, the strong coupling between the 0th harmonic oscillator and the *N* additional harmonic oscillators, and the large number of the additional harmonic oscillators make it take a very long time for the multipartite entanglement to reach a stable value. Meanwhile, the decay rate and the coupling strengths between harmonic oscillators of the *N* additional harmonic oscillators have almost no effect on the multipartite entanglement generation. Finally, in order to analyze the effect of different initial states on the multipartite entanglement generation, we discussed the entanglement generation of the *N* additional harmonic oscillators. We find that only when both the coupling strengths between the additional harmonic oscillators and the decay rate of the *N* additional harmonic oscillators take a zero value can the stable entanglement be generated, even though the stable value is very small. Our results may be verified by trapped ions in a cavity coupled to a reservoir via optical pumping [88] and provide solutions to the entanglement generation. Our results can contribute to some understanding of the entanglement generation and engineering of a system interacting with a structured environment.
